# Predictive value of faecal calprotectin in ulcerative colitis – single centre experience

**DOI:** 10.1080/07853890.2022.2082518

**Published:** 2022-05-30

**Authors:** Dora Grgić, Karlo Golubić, Marko Brinar, Željko Krznarić

**Affiliations:** a Department of Gastroenterology and Hepatology, Division of Internal medicine, University Hospital Center Zagreb, Zagreb, Croatia; b Department of Cardiology, University Hospital Centre “Sestre Milosrdnice”, Zagreb, Croatia; c School of medicine, University of Zagreb, Zagreb, Croatia

**Keywords:** Faecal calprotectin, inflammatory bowel disease, ulcerative colitis, faecal markers, biomarkers

## Abstract

**Objectives:**

Faecal calprotectin is an important biomarker used in the evaluation of inflammatory bowel disease. The aim of this study was to establish the value of faecal calprotectin concentration as a predictor of remission in ulcerative colitis and its correlation with laboratory, endoscopic and clinical findings.

**Methods:**

The single centre study included 126 adult patients with established diagnosis of ulcerative colitis consecutively visiting our Day clinic from March 2017 to March 2019. We measured serum biomarkers- CRP, haemoglobin, leukocytes and platelets. Faecal calprotectin was determined from stool, and endoscopy was performed with calculation of MAYO endoscopic subscore system (MES 0–1: remission, and MES 2–3: active disease). Clinical assessment was done by using Mayo score for ulcerative colitis (clinical Mayo score <2:remission, >5: active disease).The statistical analysis was performed using an univariate and multivariate model of disease remission prediction using logistic regression.

**Results:**

According to univariate analysis the increase of faecal calprotectin concentration by 10 ug/g is associated with an 8% decrease in probability of disease remission (OR 0.9921, *p* < .05). In the multivariate analysis, faecal calprotectin remained a significant predictor of disease remission (OR 0.9948, 95% CI 0.9914–0.9982, *p* = .0028), however, with a significant contribution of C-reactive protein (OR 0.8340, 95% CI 0.7085–0.9818, *p* = .0292). According to our model the cut off value for faecal calprotectin was 154 ug/g.

**Conclusion:**

Our results have shown that faecal calprotectin is an independent predictor of remission in UC patients. The results of our study represent real-life data from a single university centre dealing with FC as a prognostic marker in patients with UC.

**KEY MESSAGES**
- Faecal calprotectin is an independent predictor of remission in UC patients.
- Recent studies have suggested that calprotectin correlates well with endoscopic activity of inflammation but correlation of faecal calprotectin in a phase of remission hasn’t been evaluated yet.
- We have found that other inflammatory biomarkers do not correlate well with either endoscopic or clinical activity in ulcerative colitis.

## Introduction

Inflammatory bowel disease (IBD) (Crohn's disease (CD) and ulcerative colitis (UC)) are chronic diseases characterised with recurrent episodes of inflammation in the gastrointestinal tract.

CD is characterised by discontinuous regions of intestinal inflammation most frequently involving the terminal ileum and colon, but can affect any part of the gastrointestinal tract from mouth to anus. The most usual symptoms of CD are abdominal pain, weight loss and diarrhoea.The inflammation is transmural. The most common disease complications include strictures and fistula formation. The inflammatory process of ulcerative colitis (UC) is limited to the mucosa and submucosa of the colon alone with disease almost invariably involving the rectum. The most common symptoms of UC are diarrhoea, hematochezia, tenesmus and defecatory urgency [1].

Pathogenesis of IBD is very complex and multifactorial. It is considered that IBD arises as a result of inappropriate immune response to intestinal commensal organisms in individuals with genetic predisposition which consequently causes inflammation [2]. Substantial progress has been made though in understanding IBD immunopathogenesis, including the interplay between the immune system and environmental, genetic and microbial factors [3].

The diagnostic basis for IBD are endoscopic and clinical methods. Endoscopy is the gold standard for detecting inflammatory bowel disease, and it correlates with disease severity [4,5]. But it is also invasive, expensive, time-consuming, and carries the risk of complications, with perforation as one of the most dangerous ones [4]. Clinical scores that we commonly use, such as the Crohn's Disease Activity Index (CDAI) for CD and the clinical component of the Mayo score for UC are subjective and do not correlate with endoscopic findings and actual inflammatory activity. On the other hand, serum biomarkers such as C-reactive protein (CRP) are not specific because their levels are elevated in any other inflammation, not only in intestinal inflammation. Due to all mentioned above and due to minimised invasiveness, new diagnostic markers have been introduced into clinical practice.

Calprotectin is a 36-kDa calcium-binding protein that belongs to the S100 protein family. It is very stable and resistant to proteolytic degradation in faeces. Calprotectin is predominantly derived from neutrophils and has direct antimicrobial effects (it competes with bacteria over zinc, thus kills the bacteria) and a role in the innate immune response. It is also found in activated monocytes and macrophages. Since inflammation is followed by migration of these cells into the intestinal lumen, the concentration of calprotectin in stool is directly proportional to the number of neutrophils in the intestinal lumen. In inflammatory and infectious conditions, calprotectin concentration increases up to 100-fold [2,6–8].

The efficacy of faecal calprotectin as an laboratory marker in IBD diagnosis and management has been studied mostly including differentiation of IBD from irritable bowel syndrome (IBS), evaluation of endoscopic and histological activity of the disease, and prediction of disease recurrence and response to treatment [2].

Recent studies have shown that calprotectin correlates well with endoscopic activity of inflammation [7,9]. Calprotectin has been shown to be more specific and sensitive for detecting inflammation in the digestive mucosa than serologic markers [10]. However, the correlation of faecal calprotectin in a phase of remission has not yet been studied. Some authors have previously reported that faecal calprotectin tends to be more reliable in UC than in CD. This is due to the greater sensitivity for detecting lesions in the colon than in ileum and probably also because we have transmural inflammation in Crohn's disease, whereas inflammation in ulcerative colitis is confined to the mucosa [10]. Considering all this, the tendency for higher calprotectin concentration in pancolitis is clearer on UC than in colonic involvement in Crohn's disease.

In contrast to colonic involvement, the role of faecal calprotectin in ileal disease has always been controversial, as reported by some of the previous studies, but it is also important to note that recent studies have shown a good correlation between faecal calprotectin and inflammatory activity in the small intestine by capsule endoscopy and magnetic resonance [10].

Although endoscopy remains the gold standard for assessing disease activity in IBD, it is not ideal for several reasons, such as high cost, limited availability, and risk of complications, and it is often disliked by patients. Among the known serum inflammatory biomarkers, CRP is one of the most widely used, widely available, and also relatively inexpensive. It is an acute-phase reactant synthesised in the liver as an acute-phase response to various inflammatory, ischaemic, and related causes. It is considered the best biochemical marker among acute-phase mediators because it appears immediately in serum during the inflammatory stimulus, has a short half-life, and disappears rapidly after the stimuli cease. In patients with inflammatory bowel disease, an increase in CRP concentration has been reported as a good correlation with the clinical activity of the disease [11].

Considering that CRP is usually used as a general inflammatory marker, CRP concentration is not exclusive to intestinal inflammation [4]. In addition, it is estimated that approximately 15% of healthy individuals do not show a CRP response. In general, CRP level is associated with clinical disease activity in IBD, especially in Crohn's disease (CD), but it is not a specific marker for intestinal inflammation with an overall specificity of 0.49 in CD [12]. All of these limitations have led to the development of additional tests, particularly stool biomarkers, that have been shown to have higher specificity for intestinal inflammation [4,13]. Both serum biomarkers and faecal calprotectin have recently been used in the Calm trial as an effective strategy to achieve the goal of complete remission [14].

The aim of this study was to establish the value of faecal calprotectin concentration as a predictor of remission in ulcerative colitis and its correlation with laboratory, endoscopic and clinical findings.

We used a logistic regression model, namely a univariate model with faecal calprotectin and a multivariate model including clinical and laboratory parameters, and then compared their predictive value.

## Materials and methods

### Patients

The study was approved by the Ethics Committees of the University Hospital Centre Zagreb and University of Zagreb, School of Medicine. All participants signed the informed consent. Adult patients with a history of ulcerative colitis consecutively visiting our Day clinic from March 2017 till March 2019 were enrolled. The diagnosis of ulcerative colitis was based on earlier performed endoscopy.

Inclusive criteria for patients were: diagnosis of ulcerative colitis, adult age between 18 and 75. Exclusion criteria were history of malignancy, pregnancy, viral or metabolic liver disease, signs of ongoing infection- particularly gastrointestinal, chronic use of nonsteroidal anti-inflammatory drugs (at least one weekly dose), or patients unwilling to sign the consent.

## Study design

We performed a retrospective cross sectional study defining two cohorts of patients: with active and with inactive disease. Inactive disease was defined with Mayo clinical score <2, regardless of Mayo endoscopic subscore (MES) >1.

The sample size was calculated using the formula for ROC curves of prediction models under the following assumptions: Alpha 0.05, Beta 0.20, AUC 0.7, Null Hypothesis AUC 0.5, and a negative to positive ratio of 0.5. The minimal sample size was 68 patients in total [15].

## Planned interventions

### Predictors

Investigated serum biomarkers included C-reactive protein (CRP), leukocytes, platelets and haemoglobin. The values of those serum markers were determined by routine laboratory analysis.

Faecal calprotectin was measured in faecal samples collected before the preparation for colonoscopy, within the 14 days before colonoscopy. Faecal calprotectin was measured by fully automated, random access chemiluminescent immunoassay (QUANTA Flash^®^ Calprotectin, Inova Diagnostics, USA) on BIO-FLASH analyser. The test was performed after extraction of calprotectin from stool.

Laboratory markers and faecal calprotectin were taken at the same time.

### Outcomes

Clinical activity was calculated within the month before the endoscopy.

Clinical activity was calculated using the Mayo index score for UC, defined as follows: Inactive (remission): <2 points and Clinical activity: ≥5 points.

Mayo clinical score consists of 4 segments: stool frequency, rectal bleeding, findings of endoscopy and physician's global assessment. Each segment is scored from 0 to 3, and the highest score is 12, with higher scores indicating worse severity.

We decided to use Mayo clinical score for defining the disease activity because the correlation between faecal calprotectin and Mayo clinical score is less commonly evaluated than the correlation between faecal calprotectin and endoscopic activity.

Endoscopy was performed by an IBD specialist. Endoscopic activity was calculated using the Mayo endoscopic subscore (MES) for UC. MES is the most commonly used in clinical trials and clinical practice settings [2]. Endoscopic score is defined as follows: Remission (MES 0): intact vascular pattern without friability or granulation; Mild activity (MES 1): erythema, reduction of vascular pattern, or minimal granulation; Moderate activity (MES 2): pronounced erythema, granulation, friability, absent vascular pattern, bleeding with minimum trauma or absence of ulcers; Severe activity(MES 3): spontaneous bleeding or ulcerations.

According to Montreal Classification of UC, the colon is divided into the 3 segments: 1. proctitis (involvement limited to the rectum; E1); 2. left-sided colitis (involvement limited to the portion of the colorectum distal to the splenic flexure; E2); 3. extensive colitis (pancolitis; involvement extends proximal to the splenic flexure; E3).

## Methods of statistical analysis

Continuous variables are presented as medians and interquartile range. Categorical variables are displayed in their absolute frequencies and relative share.Patients were divided into groups according to indexes of activity of inflammatory bowel disease, and differences between groups (according to registered variables) were tested with Mann- Whitney test that is not dependent on distribution (for continuous variables) and with chi2 (for categorical variables). Normality of distribution of quantitative variables was examined with the Shapiro-Wilk test. Value of p less than 0.05 (bidirectional) was considered statistically significant.To determine predictive value,binary logistic regression was used [16,17].

## Results

Using the inclusion criteria outlined in Methods we studied a total of one-hundred-twenty-six (126) patients diagnosed with ulcerative colitis. Table 1 provides an overview of the clinical characteristics of UC. Overall, the age span was between 18 and 72 years, and there were 60 (47.6%) men and 66 (52.4%) women.Of the total number of patients 23 (18.3%) were diagnosed with proctitis (E1), 38 (30.2%) with left-sided-colitis (E2), and 65 (51.6%) with pancolitis (E3).Also, of the total number of patients 16 (12.7%) were receiving corticosteroids, 94 (74.6%) aminosalicylates, 49 (38.9%) biologic therapy, and 16 (12.7%) immunosuppressive. Table 2 presents the values of the different biological markers studied.According to MAYO clinical score for ulcerative colitis patients were divided into two groups- active disease and remission. Of the total number of patients 56 (44.4%) had active disease (Mayo score> 2), and 70 (55.6%) were in remission (Mayo score 2 or <2). In active disease the median faecal calprotectin concentration was 382,5 ug/g (197.5–1191.5) and in remission the median faecal calprotectin concentration was 44 ug/g (18–90). Calprotectin was significantly higher in patients with active disease (*p* < .001).

**Table 1.** Epidemiological and clinical characteristics of the patients.

Variable	*N*	(%)
Female sex	66	52.40
Age (years)	40	29–52
Extension		
1	23	18.30
2	38	30.20
3	65	51.60
Age at diagnosys (years)	31	22–43
Disease duration (months)	72	24–120

**Table 2.** Median values of the biological markers studied.

	Active disease	Interquartile range	%	Remission	Interquartile range	%	*p* Value	test
N	56		44,4	70		55,6		
Faecal calprotektin (μg/g)	382.5	197.5–1191.5		44	18–90		<.001	Mann-Whitney test
Leukocytes (x10^9^)	8.2	6.35–11.15		6,05	4.9–7.6		<.001	Mann-Whitney test
Haemoglobin (g/L)	134	123–145.5		142,5	131–152		.002	Mann-Whitney test
Platelet count (x10^9^)	293.5	250–362.5		235	205–281		<.001	Mann-Whitney test
CRP (mg/L)	7.1	2.3–20		1,25	0.6–2.8		<.001	Mann-Whitney test

Observing the other biomarkers we found statistically significant differences in value of leukocytes whose mean value was 8.2 × 10^9^/L in active disease (6.35–11.15) vs 6.05 × 10^9^/L (4.9–7.6) in remission (reference interval for leukocyte level is 3.4–9.7 × 10^9^/L). Haemoglobin- mean value in active disease 134 g/L/123–145.5) vs 142.5 g/L (131–152) in remission (reference interval for haemoglobin 138–175 g/L); platelets – mean value in active disease 239.5 × 10^9^/L (250–362.5) vs 235 × 10^9^/L (205–281) in remission (reference value for platelets is 158–424 × 10^9^/L). Only C-reactive protein was significantly different between the two groups. Mean value of C- reactive protein was 7.1 mg/L (2.3–20) in active disease vs 1.25 mg/L (0.6–2.8) in remission (reference interval for C-reactive protein is <5 mg/L).

Also, according to the extension of disease, in proctitis the percentage of patients in remission was 80%, and with other localizations (left-sided colitis, pancolitis) the percentage of patients in remission was 50%.

We constructed an univariate and multivariate model of disease remission prediction using logistic regression. According to our univariate model the increase of faecal calprotectin concentration by 10 ug/g is associated with an 8% decrease in disease remission (OR 0.9921, 95% CI 0.9885–0.9957) *p* < .05) Table 3.

**Table 3.** Univariable and multivariable models.

Variable	OR	95% CI		*p* Value
		low	high	
Faecal calprotectin	0.9921	0.9885	0.9957	.0000
CRP	0.7817	0.6947	0.8796	<.0001
SEX	0.9580	0.4740	1.9760	.9048
Age	0.9967	0.9708	1.0233	.8056
Haemoglobin	1.0238	1.0017	1.0465	.0350
Leukocytes	0.6986	0.5908	0.8260	<.0001
Multivariable model				
Age	1.004	0.9646	1.0449	.8463
Sex	0.5986	0.1798	1.9929	.4030
Faecal calprotectin	0.9948	0.9914	0.9982	.0028
Leukocytes	1.0101	0.7495	1.3613	.9473
Haemoglobin	1.0473	0.9980	1.0990	.0604
Plateletes	0.9987	0.9906	1.0069	.7571
CRP	0.8340	0.7085	0.9818	.0292

**Figure 1.**
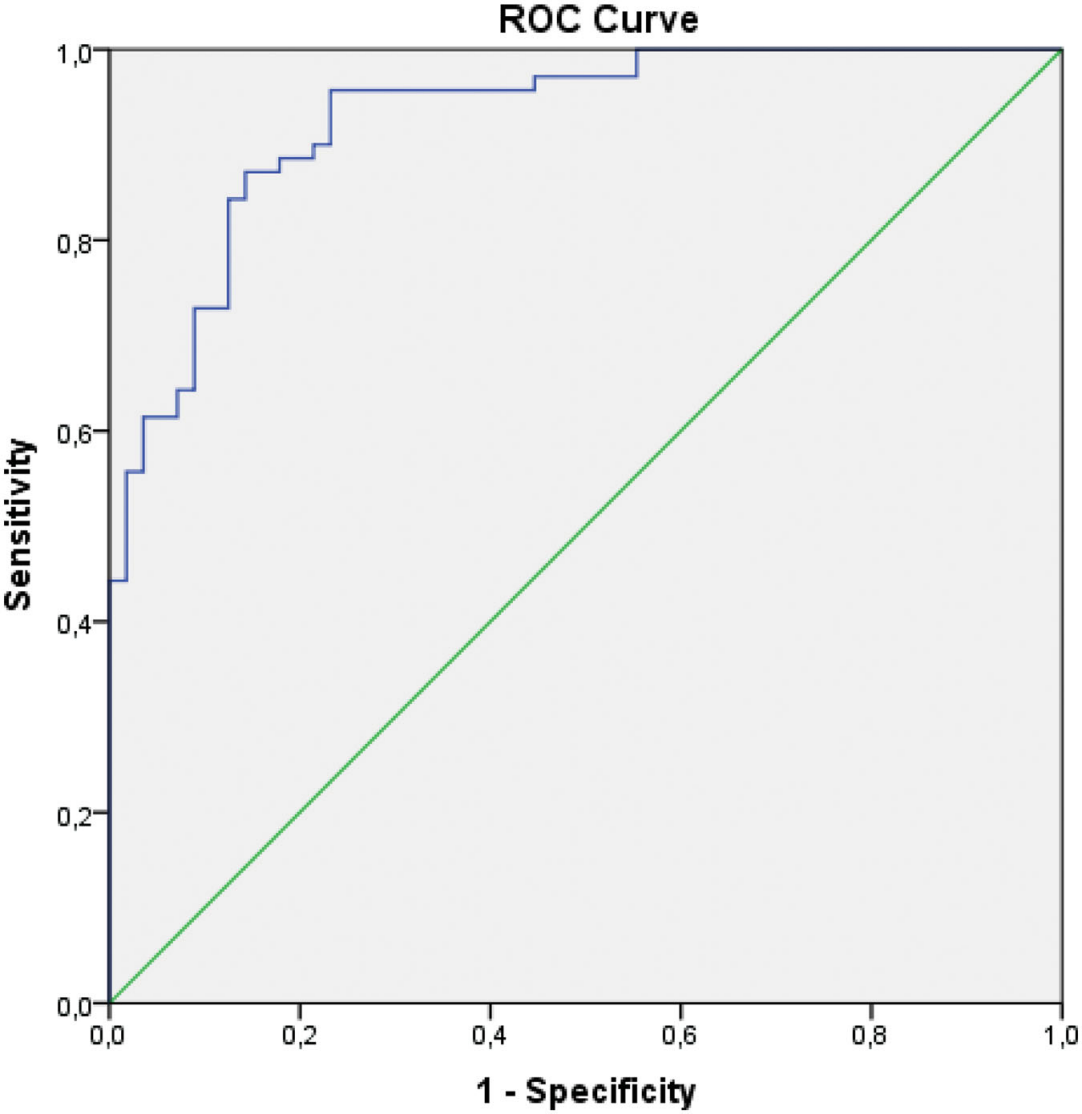
Receiver operating curve comparison of the univariable models.

The univariable model ROC curves are compared in Figure 1.

In the multivariate model, faecal calprotectin remained a significant predictor of disease remission (OR 0.9948, 95% CI 0.9914–09982, p 0.0028), however, with a significant contribution of C-reactive protein (OR 0.7085, 95% CI 0.7085–0.9818, p 0.0292) Table 3.

In our model the area under the curve was 0.912, with specificity 0.768 and sensitivity 0.929, while the positive and negative predictive values were 79% and 86.7%, respectively. The classification accuracy was 0.857, information score 0.518, F-measure 0.878, Precision 0.833, Recall 0.923, Brier score 0.25, and the Marrhews correlation coef 0.713.

**Figure 2.**
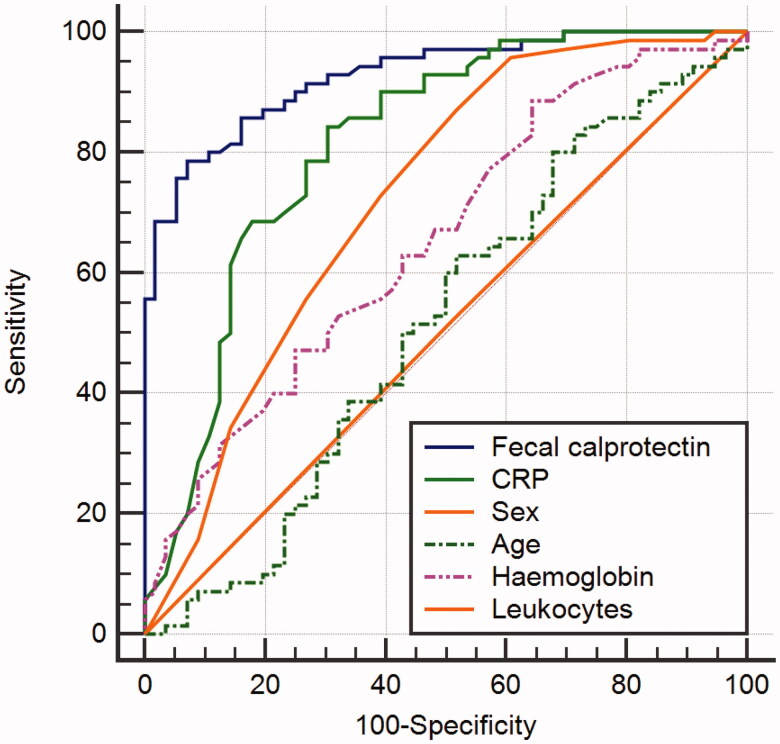
Receiver operating curve analysis (ROC) of faecal calprotectin.

The ROC curve was constructed for the logistic regression model- Figure 2.

**Figure 3.**
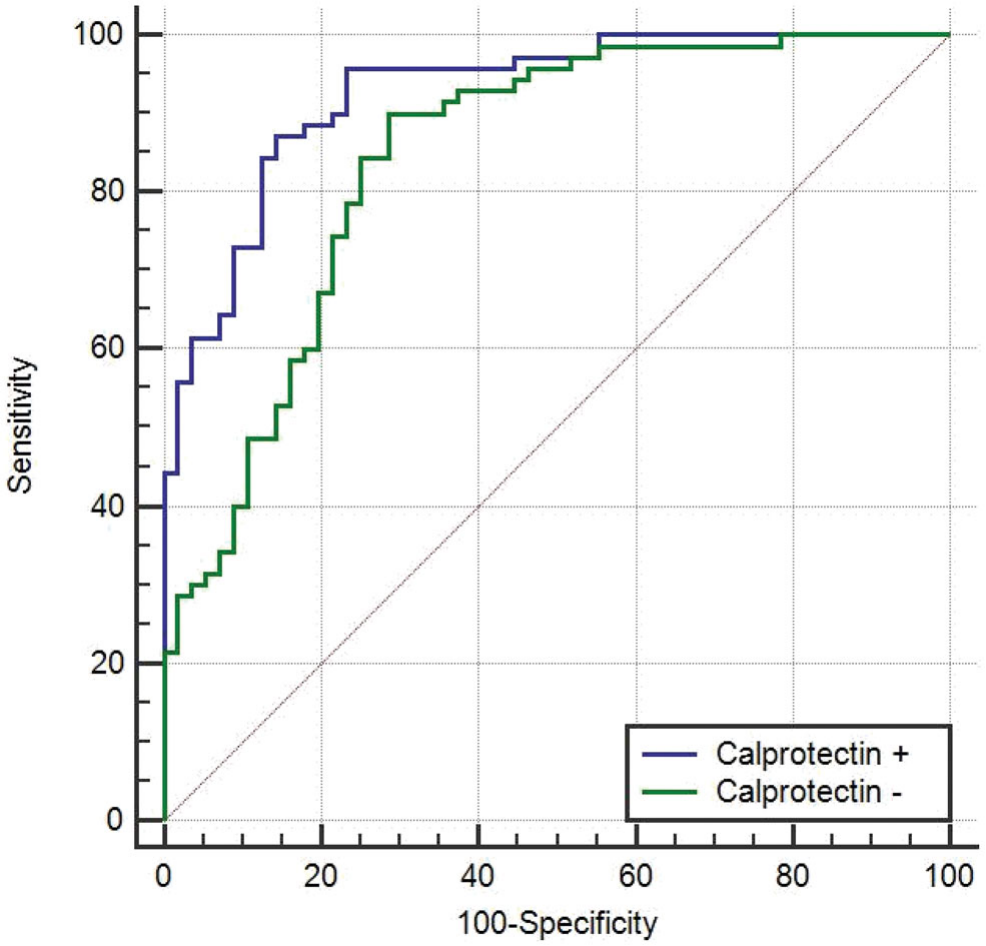
Receiver operating curve comparison of multivariable models with (+) and without (-) calprotectin.

In comparison, the multivariable model containing faecal calprotectin was better at predicting remission then the model without calprotectin, AUC 0.927 (95% CI 0.867 to 0.966) vs 0.843 (95% CI 0.768 to 0.902) respectively, *p* = .0009, Figure 3.

Fcal had significantly greater area under the curve than other studied variables Table 4.

**Table 4.** Analysis of ROC curves of univariable models.

Variable	AUC	SE^a^	95% CI^b^
Faecal calprotectin	0.928	0.0216	0.867 to 0.966
	Pairwise comparison of ROC curves		
	vs.		*p* Value
	CRP		.0046
	SEX		<.0001
	Age		<0.0001
	Haemoglobin		< 0.0001
	Leukocytes		< 0.0001
CRP	0.815	0.0402	.736 to .878
SEX	0.505	0.0451	.415 to .596
Age	0.517	0.0534	.426 to .607
Haemoglobin	0.654	0.0489	.564 to .737
Leukocytes	0.724	0.0467	.637 to .800

## Discussion

Colonoscopy is currently considered the standard test for diagnosis and monitoring of IBD. However, this procedure is invasive, expensive, and often requires analgosedation and demanding preparation. Over the past two decades, faecal biomarkers such as FC have emerged as potential replacements for endoscopy. Faecal biomarkers have certain advantages as they can be easily collected and they provide the ability to monitor disease on a regular basis [10].

The results of our study represent real data from a single university centre investigating the role of FC and its correlation with laboratory, endoscopic, and clinical findings in predicting remission in patients with UC. The results of this study show that faecal calprotectin is a very accurate predictor of endoscopic activity in ulcerative colitis. Recent studies have shown that calprotectin correlates well with endoscopic inflammatory activity, but the correlation of faecal calprotectin in a phase of remission has not been studied, and endoscopy has still remained the gold standard for assessing inflammatory activity in IBD [18].

In our study, we chose to use the Mayo clinical score to define disease activity because the correlation between faecal calprotectin and the Mayo clinical score is less frequently studied than the correlation between faecal calprotectin and endoscopic activity. The number of patients who were endoscopically active (MES 2) but were in remission according to the clinical Mayo score is very small (7 patients out of a total of 126) and did not affect our results.

Calprotectin is highly valued as a marker to distinguish between organic and functional diseases-it was described in its early days as a marker to distinguish between irritable bowel syndrome and inflammatory bowel disease [14,18]. Consistent with previous publications, we found statistically significant differences in faecal calprotectin concentrations according to endoscopic activity. We also examined other serum biomarkers to assess endoscopic activity in CED, but only CRP showed statistical significance, whereas other biomarkers were inappropriate for endoscopic activity [19]. CRP was used before to distinguish between quiescent and active disease, but in general, the correlation between CRP and endoscopic activity is lower than that between faecal markers and activity [12,18]. Of the publications to date, one of note is that of Tibble *et al.* who studied 220 adult patients to determine inflammatory bowel disease and irritable bowel syndrome. They used faecal calprotectin with a discrimination limit of 10 mg/L. Sensitivity was 82% and specificity was 83%. The same group of authors measured faecal calprotectin concentration in 602 adults: In the study, 44% of the total population was classified as organic disease and 56% as functional disorder. Sensitivity and specificity of faecal calprotectin were 89% and 79%, respectively [20]. Gisbert *et al.* concluded in a 2009 study that faecal calprotectin and lactoferrin determination may be useful in predicting impending clinical relapse [21]. Also, in 2008, Xiang *et al.* showed that the concentration of faecal calprotectin was significantly higher in patients with active ulcerative colitis than in patients with inactive disease and the control group [22]. Roseth and co-workers proved that faecal calprotectin concentration correlates well with endoscopic and histologic assessment of disease activity in adults and children with IBD [23]. Also, many other studies concluded that faecal calprotectin discriminates more accurately than other faecal markers (lactoferrin, polymorphonuclear neutrophil elastase) between irritable bowel syndrome and organic bowel disease. Finally, Samant *et al.* in 2015. published the work concluding that faecal calprotectin correlated better with disease activity than serum biomarkers CRP and ESR [24].

In 2015, the Selective Therapeutic Targets in Inflammatory Bowel Disease (STRIDE) guidelines were published with the goal of providing consensus recommendations on which targets should be prioritised in clinical practice [25].

The researchers concluded that at UC, disease activity should be assessed by the disappearance of clinical symptoms and by objective inflammatory parameters assessed endoscopically with the Mayo endoscopic scoring system (MES 0.1) [25]. The committee determined that there was no direct evidence of an association between histologic remission, biomarkers, and imaging studies and improved treatment outcomes. However, the use of biomarkers as surrogates for specific targets may have clinical value [26]. The 2015 observational study by Burri and colleagues included 41 patients with ulcerative colitis and measured activity using faecal calprotectin and the clinical activity index (CAI). The conclusion was that faecal calprotectin was similarly useful as CAI to monitor disease activity of ulcerative colitis during medical treatment [27].

Our study is subject to limitations because it is a single-centre study that is susceptible to selection bias. Also, the limited number of participants is a common limitation in studies investigating multiple biomarkers for a given disease. Blood samples for serum biomarkers were collected only once, as were stool samples for faecal calprotectin concentrations, which may have introduced bias from individual variation. All patients were Caucasians recruited from the same geographical area, which drew away possible ethnic bias [28]. Participants also underwent identical diagnostic procedures performed by the same group of investigators, so direct comparisons were entirely justified.

## Conclusion

In summary, we have found and confirmed that faecal calprotectin is a highly reliable predictor of endoscopic and clinical activity in ulcerative colitis patients. We have also found that other inflammatory biomarkers do not correlate well with neither endoscopic nor clinical activity in ulcerative colitis. There is a very good correlation between the value of faecal calprotectin and clinical remission and that is the highlight of our study.

## Data Availability

The data that support the findings of this study are available from the corresponding author, upon reasonable request.

## References

[1] Iskandar HN, Ciorba MA, Biomarkers in inflammatory bowel disease: current practices and recent advances. Transl Res. 2012;159(4):1570–325. 10.1016/j.trsl.2012.01.001 PMC3308116 22424434

[2] Khaki-Khatibi F, Qujeq D, Kashifard M, et al. Calprotectin in inflammatory bowel disease. Clin Chim Acta. 2020;510:556–565. 32818491 10.1016/j.cca.2020.08.025 PMC7431395

[3] de Souza HS, Fiocchi C. Immunopathogenesis of IBD: current state of the art. Nat Rev Gastroenterol Hepatol. 2016;13(1):13–27. 26627550 10.1038/nrgastro.2015.186

[4] Mosli MH, Zou G, Garg SH, et al. C-Reactive protein, fecal calprotectin, and stool lactoferrin for detection of endoscopic activity in symptomatic inflammatory bowel disease patients: a systematic review and Meta-Analysis. Am J Gastroenterol. 2015;110(6):802–819. 25964225 10.1038/ajg.2015.120

[5] Yamamoto T, Shimoyama T, Umegae S, et al. Endoscopic score vs fecal biomarkers for predicting relapse in patients with ulcerative colitis after clinical remission and mucosal healing. Clin Transl Gastroenterol. 2018;9(3):136. 29491393 10.1038/s41424-018-0006-7 PMC5862153

[6] Manceau H, Chicha-Cattoir V, Puy H, et al. Fecal calprotectin in inflammatory bowel diseases: update and perspectives. Clin Chem Lab Med. 2017;55(4):474–483. 27658156 10.1515/cclm-2016-0522

[7] Sipponen T, Kolho KL. Fecal calprotectin in diagnosis and clinical assessment of inflammatory bowel disease. Scand J Gastroenterol. 2015;50(1):74–80. 25523558 10.3109/00365521.2014.987809

[8] Mumolo MG, Bertani L, Ceccarelli L, et al. From bench to bedside: fecal calprotectin in inflammatory bowel diseases clinical setting. World J Gastroenterol. 2018;24(33):3681–3694. 30197475 10.3748/wjg.v24.i33.3681 PMC6127662

[9] Kostas A, Siakavellas SI, Kosmidis C, et al. Fecal calprotectin measurement is a marker of short-term clinical outcome and presence of mucosal healing in patients with inflammatory bowel disease. WJG. 2017;23(41):7387–7396. 29151692 10.3748/wjg.v23.i41.7387 PMC5685844

[10] Jusué V, Chaparro M, Gisbert JP. Accuracy of fecal calprotectin for the prediction of endoscopic activity in patients with inflammatory bowel disease. Digest Liver Dis. 2018;50(4):353–359. 10.1016/j.dld.2017.12.022 29396129

[11] Vucelić B, Krznarić Z, Sentić M, et al. Vrijednost C-reaktivnog proteina u procjeni aktivnosti ulceroznog kolitisa i crohnove bolesti [value of C-reactive protein in the evaluation of activity in ulcerative colitis and crohn's disease]. Lijec Vjesn. 1990;112(9-10):281–284. 2093781

[12] Chen J-M, Liu T, Gao S, et al. Efficacy of noninvasive evaluations in monitoring inflammatory bowel disease activity: a prospective study in China. WJG. 2017;23(46):8235–8247. 29290660 10.3748/wjg.v23.i46.8235 PMC5739930

[13] Lewis JD. The utility of biomarkers in the diagnosis and therapy of inflammatory bowel disease. Gastroenterology. 2011;140(6):1817–1826. 21530748 10.1053/j.gastro.2010.11.058 PMC3749298

[14] Colombel JF, D'haens G, Lee WJ, et al. Outcomes and strategies to support a treat-to-target approach in inflammatory bowel disease: a systematic review. J Crohns Colitis. 2020;14(2):254–266. 31403666 10.1093/ecco-jcc/jjz131 PMC7008150

[15] Hanley JA, McNeil BJ. The meaning and use of the area under a receiver operating characteristic (ROC) curve. Radiology. 1982;143(1):29–36. 7063747 10.1148/radiology.143.1.7063747

[16] Peduzzi P, Concato J, Kemper E, et al. A simulation study of the number of events per variable in logistic regression analysis. J Clin Epidemiol. 1996;49(12):1373–1379. 8970487 10.1016/s0895-4356(96)00236-3

[17] Rainey C, McCaskey K. Estimating logit models with small samples. Condit Accepted Polit Sci Res Methods. 2021;9:549–564

[18] Vernia F, Di Ruscio M, Stefanelli G, et al. Is fecal calprotectin an accurate marker in the management of Crohn's disease? J Gastroenterol Hepatol. 2020;35(3):390–400. 31795013 10.1111/jgh.14950

[19] Takenaka K, Tominaga K, Kanazawa M, et al. Endoscopic score vs blood cell indices for determining timing of immunomodulator withdrawal in quiescent ulcerative colitis. Sci Rep. 2019;9(1):17751. 31780764 10.1038/s41598-019-54369-7 PMC6882869

[20] Tibble J, Teahon K, Thjodleifsson B, et al. A simple method for assessing intestinal inflammation in crohn's disease. Gut. 2000;47(4):506–513. 10986210 10.1136/gut.47.4.506 PMC1728060

[21] Gisbert JP, Bermejo F, Perez-Calle JL, et al. Fecal calprotectin and lactoferrin for the prediction of inflammatory bowel disease relapse. Inflamm Bowel Dis. 2009;15(8):1190–1198. 19291780 10.1002/ibd.20933

[22] Xiang JY, Ouyang Q, Li GD, et al. Clinical value of fecal calprotectin in determining disease activity of ulcerative colitis. WJG. 2008;14(1):53–57. 18176961 10.3748/wjg.14.53 PMC2673391

[23] Roseth AG, Aadland E, Jahnsen J, et al. Assessment of disease activity in ulcerative colitis by faecal calprotectin, anovel granulocyte marker protein. Digestion. 1997;58(2):176–180. 9144308 10.1159/000201441

[24] Samant H, Desai D, Abraham P, et al. Fecal calprotectin and its correlation with inflammatory markers and endoscopy inpatients from India with inflammatory bowel disease. Indian J Gastroenterol. 2015;34(6):431–435. 26589229 10.1007/s12664-015-0608-x

[25] Pabla BS, Schwartz DA. Assessing severity of disease in patients with ulcerative colitis. Gastroenterol Clin North Am. 2020;49(4):671–688. 33121688 10.1016/j.gtc.2020.08.003 PMC7510557

[26] Gonczi L, Bessissow T, Lakatos PL. Disease monitoring strategies in inflammatory bowel diseases: what do we mean by "tight control"? “?. World J Gastroenterol. 2019;25(41):6172–6189. 31749591 10.3748/wjg.v25.i41.6172 PMC6848014

[27] Burri E, Beglinger C, von Felten S, et al. Fecal calprotectin and the clinical activity index are both useful to monitor medical treatment in patients with ulcerative colitis. Dig Dis Sci. 2015;60(2):485–491. 25344905 10.1007/s10620-014-3383-0

[28] Kurkowska-Jastrzebska I, Karlinski MA, Błazejewska-Hyzorek B, et al. Carotid intima media thickness and blood biomarkers of atherosclerosis in patients after stroke or myocardial infarction. Croat Med J. 2016;57(6):548–557. 28051279 10.3325/cmj.2016.57.548 PMC5209935

